# CE-UV/VIS and CE-MS for monitoring organic impurities during the downstream processing of fermentative-produced lactic acid from second-generation renewable feedstocks

**DOI:** 10.1186/s13036-016-0027-2

**Published:** 2016-05-19

**Authors:** Hendrik Laube, Frank-Michael Matysik, Andreas Schmidberger, Kerstin Mehlmann, Andreas Toursel, Jana Boden

**Affiliations:** Department of Bioengineering, Leibniz-Institute for Agricultural Engineering (ATB), Max-Eyth-Allee 100, Potsdam, 14469 Germany; Institute of Analytical Chemistry, Chemo- and Biosensors, University of Regensburg, Regensburg, Germany; ICA Boden-Haumann-Mainka, Engineering Society for Chemical Analysis, Langen, Hessen Germany

**Keywords:** Bio-based chemicals, High volume chemicals, Lactic acid, Non-targeting screening, Organic impurity monitoring, Downstream processing, Capillary electrophoresis, Mass spectrometry, Pyroglutamic acid

## Abstract

**Background:**

During the downstream process of bio-based bulk chemicals, organic impurities, mostly residues from the fermentation process, must be separated to obtain a pure and ready-to-market chemical. In this study, capillary electrophoresis was investigated for the non-targeting downstream process monitoring of organic impurities and simultaneous quantitative detection of lactic acid during the purification process of fermentatively produced lactic acid. The downstream process incorporated 11 separation units, ranging from filtration, adsorption and ion exchange to electrodialysis and distillation, and 15 different second-generation renewable feedstocks were processed into lactic acid. The identification of organic impurities was established through spiking and the utilization of an advanced capillary electrophoresis mass spectrometry system.

**Results:**

A total of 53 % of the organic impurities were efficiently removed via bipolar electrodialysis; however, one impurity, pyroglutamic acid, was recalcitrant to separation. It was demonstrated that the presence of pyroglutamic acid disrupts the polymerization of lactic acid into poly lactic acid. Pyroglutamic acid was present in all lactic acid solutions, independent of the type of renewable resource or the bacterium applied. Pyroglutamic acid, also known as 5-oxoproline, is a metabolite in the glutathione cycle, which is present in all living microorganisms. pyroglutamic acid is found in many proteins, and during intracellular protein metabolism, N-terminal glutamic acid and glutamine residues can spontaneously cyclize to become pyroglutamic acid. Hence, the concentration of pyroglutamic acid in the lactic acid solution can only be limited to a certain amount.

**Conclusions:**

The present study proved the capillary electrophoresis system to be an important tool for downstream process monitoring. The high product concentration encountered in biological production processes did not hinder the capillary electrophoresis from separating and detecting organic impurities, even at minor concentrations. The coupling of the capillary electrophoresis with a mass spectrometry system allowed for the straightforward identification of the remaining critical impurity, pyroglutamic acid. Although 11 separation units were applied during the downstream process, the pyroglutamic acid concentration remained at 12,900 ppm, which was comparatively high. All organic impurities found were tracked by the capillary electrophoresis, allowing for further separation optimization.

**Electronic supplementary material:**

The online version of this article (doi:10.1186/s13036-016-0027-2) contains supplementary material, which is available to authorized users.

## Background

The rising demand for chemicals and the finite nature of fossil resources, combined with the general concern about the impact on climate change, have created a growing need for sustainable, future-oriented strategies [1]. In the medium to long term, biomass is the only regenerative carbon source that is available worldwide [2]. The conversion of biomass into biochemicals that have the same quality, i.e., purity, than their petrochemical counterparts at a competitive price is the great challenge of the biotechnology industry in the 21^st^ century. As the most economic resource of carbon and nitrogen, agricultural residues, also known as 2^nd^-generation renewable resources, are used in the upstream process (USP) of bio-based chemicals [3, 4]. However, different types of impurities can be present during the manufacturing process of biochemicals, originating from the feedstock, by-products, intermediates, degradation products, reagents, and metabolites from bacteria [5, 6]. These impurities, even at very low concentrations, can dramatically alter product characteristics such as color, odor or polymer stability. Furthermore, the fact that the composition of biomass is subject to regional and seasonal change creates new challenges for the production of bio-based chemicals [7, 8].

Lactic acid (LA) is widely used in industry as a building block monomer for the polymerization of poly(LA) (PLA), also known as bioplastic. However, it is necessary to utilize highly purified LA as a ready to drop-in chemical or as a substituent for petrochemical-based LA. At the end of the downstream process (DSP), only impurities that have properties similar to the product are present, which results in a need for a highly selective capillary electrophoresis (CE) separation and detection method. A robust and reliable CE method has recently been developed, allowing for the detection of organic impurities during the downstream processing of 2^nd^-generation renewable feedstocks into LA, and LA obtained from corn, a 1^st^-generation renewable resource, became widely available when Nature Works LLC (Blair, NE, USA) began production of an estimated 180,000 annual tones in 2001 [9].

Since then, many 2^nd^-generation renewable resources have been converted by fermentation into LA in small-scale experiments [10–12]. In contrast, only a few studies have been conducted at a larger scale using pilot plants with a complete DSP [13], and such studies focused on the ion concentration, rather than organic impurities, during LA production [14–18]. With the steady demand for increased production efficiency, a holistic view of the entire process from resource and bacteria to pure bio-LA and final PLA is mandatory.

The identification, separation, and the subsequent exclusion of impurities during the DSP is an important step towards the pure and ready-to-market PLA. CE is a versatile and fast method for separation. In combination with an universal detection method, such as mass spectrometry (MS), the CE allows the detection of numerous compounds, which can be found in the biotechnology industry [19, 20]. High electrical field strength in combination with short capillaries enable the rapid separation of complex sample material [21, 22].

Here, a cradle to grave analysis of a process for LA from 15 different 2^nd^-generation renewable resources and 5 different LA bacteria by means of an analytical method for monitoring organic impurities during the DSP of LA is presented. The focus of the study is on process monitoring of the conversion of 2^nd^-generation resources into LA that is of high purity and ready for further polymerization. The target was to track critical impurities during the DSP and to identify them by means of spiking or upgrading the CE system by an advanced CE-MS setup. The critical impurities found were evaluated according to their potential to interrupt the polymerization reaction. The origins of the critical impurities were then identified; the necessary precautions to prevent their appearance are discussed.

## Results and discussion

### LA DSP benchmarks

**Fig. 1:**
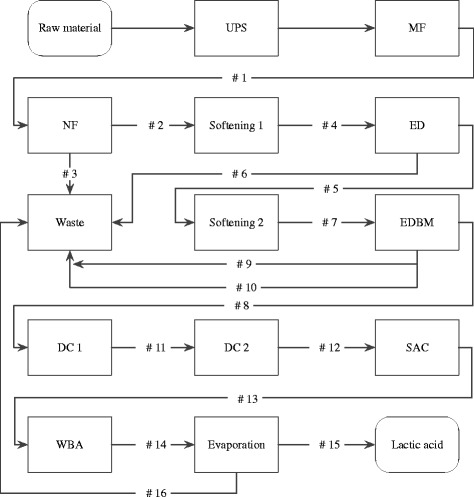
Block diagram of the DSP

**Fig. 2:**
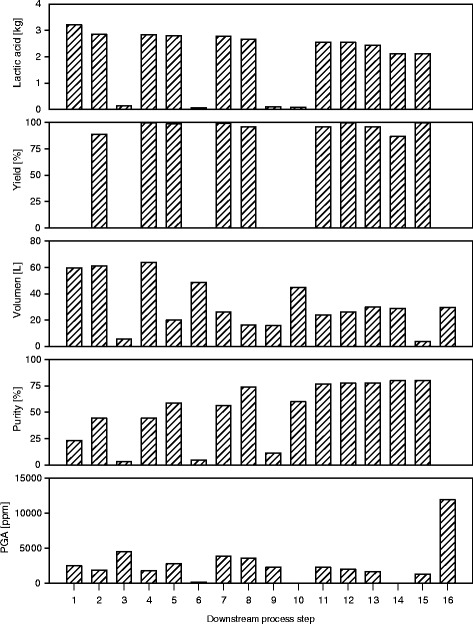
Block diagram of LA amount, yield, purity and volume of the process stream for the entire DSP

**Fig. 3:**
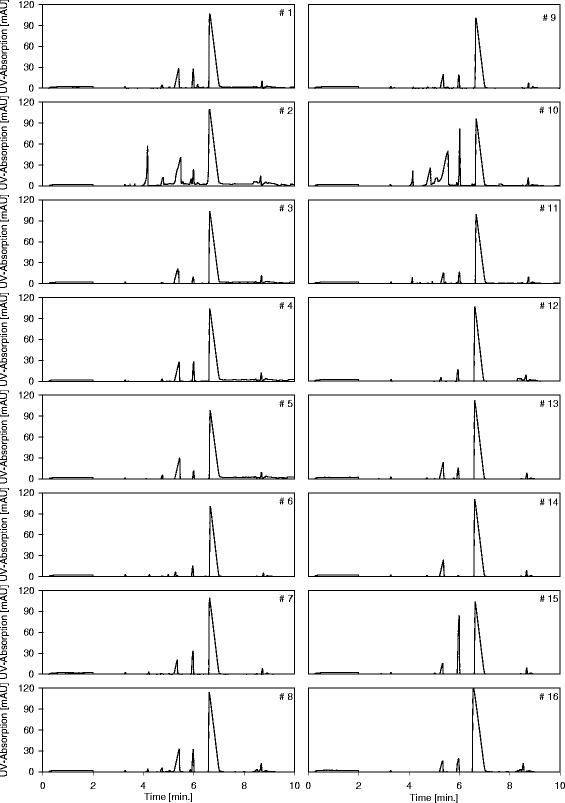
Electropherograms of LA produced from different resources

The purpose of the DSP presented in Fig. 1 was to purify, convert sodium lactate into LA and concentrate the LA produced. The DSP was evaluated according to benchmarks: LA amount, yield, purity and overall process stream volume. These parameters are provided in Fig. 2. For a better understanding of the following discussion the numeration #1 – #15 has been introduced. The Figs. 1, 2, and 3 are relating to this numeration of the samples taken from the DSP. The amount of lactate obtained from the fermenter was 3.2 kg from 59.7 L of aqueous fermentation broth. The DSP removed the majority of organic impurities, converted the lactate into LA and removed excess water. The resulting amount of LA was 2.12 kg in 3.6 L of concentrated LA aqueous solution.

The highest impact on LA purity was by the NF (#2), at the beginning of the DSP. As the purpose of filtration is the removal of macromolecules and coarse particles, it therefore greatly enhances purity, which was clearly indicated by the increase in purity of 21 %.

The precipitation of bivalent ions onto the electrodialysis membranes was prevented by two softening steps using chelating resins (#4 and #7) in between the ED and the EDBM. The ED and the EDBM enhanced the purity of the LA stream by 14 % and 17 %, respectively. In addition, the ED (#8) concentrated the sodium lactate in the product stream threefold from 44,510 to 140,350 ppm, and the EDBM (#13) converted the sodium lactate into its free acid, LA.

During the process, the LA is contained in the so-called product stream. An optimal separation unit converts the ingoing product stream, or feed stream, into an outgoing product stream with enhanced purity and an outgoing waste stream with minor purity. Although the waste stream can contain a minor concentration of the product, it always contains the major concentration of the impurities and/or unwanted ions.

There are four waste streams (#3, #6, #9, and #10) located in the DSP. The course of the purity of LA in the waste stream reflected the nature of the separation mechanism described above. The LA purity was low (23 % and 44 %) in the first separation steps, MF (#1) and NF (#2); therefore, many impurities were contained in the first two waste streams. Waste streams #3 and #6 had a purity of 3.2 % and 4.8 %, respectively. Thus, the LA purity was gradually increased by the DSP. The increasing purity of the LA stream was consistent with the increasing purity of the later waste streams. Waste streams #9 and #10 had a purity of 11.4 % and 60 %, respectively. The overall increase in LA purity for the entire DSP was 57 %, with an initial purity after the MF (#1) of 23 % and a final purity of 75 %.

In the last waste streams of the DSP, contaminants with similar characteristics to LA were removed. However, the separations caused LA losses because the LA was present at a high concentration and molecules with similar characteristics lead to an equal separation behavior of impurities and LA in the separation unit. This behavior was recorded by the yield of the WBA (#14), at 86.7 %. This was the lowest yield and hence the highest LA loss found in the DSP. Because lactate is negatively charged, a certain loss of LA (13.3 %) on the WBA was expected. The second lowest yield was found in the NF (#2), at 88.89 %, which was due to the remaining retentate, the dead volume of the filtration unit. The rest of the employed separation units had yields >95.9 %.

The course of LA purity in the EDBM provides a good example of how the separation mechanism described above affects separation. The lactate feed stream (#7) was converted into a purified LA product stream (#8), a salt (sodium lactate) waste stream (#9), and a base (caustic soda) waste stream (#10). Both waste streams contained low amounts of LA; the salt waste stream (6,700 ppm) had a concentration of LA three times higher than the base waste stream (1,900 ppm), though the purity in the salt waste stream (11 %) was below that of the base waste stream (60 %).

The differences in purity can be explained by the electropherogram (EPG) generated by the CE system shown in Fig. 3. In the EPG, several unknown peaks of impurities are detected along with the LA.

The EPG of the feed stream (#7) shows the lactate peak at 7 min, a major impurity peak at 6 min, and a fraction of minor impurity peaks between 3 min 30 s and 5 min 30 s. Some of the organic impurities were successfully identified through spiking (see Additional file 1: Figure S11). The proximity of the major impurity peak to the lactate peak indicates similar ion mobility. The salt waste stream (#9) is the reduced feed stream (#7); it did not pass any membrane during the electrodialysis process. The EPG of the salt waste stream (#9) leaving the EDBM shows a small lactate peak, a decreased peak width of the major impurity peak, and constant minor impurity peaks.

The high lactate concentrations in the feed compartment led to the migration of small concentrations of lactate into the base compartment in the EDBM. The EPG of the base waste stream (#10) shows those small amounts of migrated lactate concentration. Apparently, no other impurities migrated though the cation-selective membrane. The sodium lactate was converted into its free acid form and left the EDBM as LA. In the EPG of the LA product stream (#8), the LA peak is present, and the minor impurity peaks are absent. However, the major impurity peak in the proximity of the LA peak is present with the same intensity as the LA peak. The residual impurity obviously had similar characteristics as the product and was therefore able to pass the anion-selective membrane as the LA did. Thus, the selectivity of the membrane was not high enough to successfully retain the impurity in the feed compartment. This shows that the process of impurity removal, which occurred in the EDBM and throughout the DSP, can be detected by the CE method.

After the EDBM, colorants were removed by the DC. It is important to mention that the first column was overloaded during the DC process; therefore, the same DC resin was applied twice (#11 and #12).

The evaporation step (#15) removed the excess water, leaving the concentrated LA product. The EPG of the condensate (#15) and concentrate (#16) shows that the impurity could also not be separated from the LA through the distillation process [23].

### CE-MS for the identification of the critical impurity

To determine the exact structure and identity of the critical impurity, coupling of the CE with a mass spectrometer (MS) was necessary. This process requires expert knowledge, as the background electrolyte (BGE) used with the CE system cannot be applied in CE-MS. The ignition of the BGE in the MS capillary would generate male volatile compounds, which then become deposited in the capillary, leading to dis-functioning of the MS.

To successfully couple CE and MS, the formerly employed BGE had to be substituted for an aqueous BGE. However, this aqueous BGE did not have the same efficiency in separating the complex sample matrix as the organic BGE. Therefore, ammonium acetate was added to the water to substitute for the loss of the organic BGE. With the resulting CE-MS system, the identification of the critical impurity was determined as described below.

The aqueous BGE was established using an aqueous solution containing 25 mM ammonium acetate adjusted to pH 8.6 with ammonia. The sample was diluted 1 to 5 with the aqueous BGE, and the separation was carried out using a voltage of 25 or 30 kV, a hydrodynamic injection time of 20 s and a negative ESI-MS mode. Under these conditions, an impurity with a molecular mass (m/z) of 128.043 could be separated from the LA in the process sample leaving the anion exchanger (#14) (see Additional file 1: Figure S1). The mass of the unknown compound is 128.0422 (see Additional file 1: Figure S2). A list of possible molecular formulas with the corresponding mass deviations and mSigma values (fit of isotopic pattern) was generated (see Additional file 1: Table S1). The best agreement concerning mass accuracy and fit of the isotopic pattern was clearly obtained for the molecular formula C_5_H_6_NO_3_. Some of the other formulas were excluded based on chemical plausibility. The isotopic pattern shows very good agreement between the measured and calculated isotopic patterns (see Additional file 1: Figure S3).

The CE migration behavior of the unknown compound suggests the presence of a carboxylic group that is completely dissociated under the experimental conditions used. The m/z value of 128.0422 should therefore be assigned to the deprotonated [M-H]^−^ species. Among several possible structures, pyroglutamic acid (PGA) was considered to be the most probable compound in the given context. Further experiments were undertaken to prove this assumption.

The electrophoretic migration times of the impurity (in the real sample) and a standard solution of 1 mM PGA in the LA matrix (see Additional file 1: Figure S1) were assessed. Repetitive measurements (*n* = 10) were performed to determine the migration times and the corresponding standard deviations for the impurity in the real sample (#14) and for the PGA standard solution. The results were as follows: the impurity in the real sample (#14) had a mean of migration time of 102 s (standard deviation, 3 s), and the 1 mM PGA standard solution in the LA matrix had a mean of migration time of 104 s (standard deviation, 3 s).

The mass spectrum of a PGA standard was recorded by direct sample injection (see Additional file 1: Figure S5). The mass accuracy and the signal pattern are both in perfect agreement with the data recorded for the impurity in the sample (#14).

As additional proof for the assignment of the impurity in sample (#14) to PGA, NMR experiments were performed (see Additional file 1: Figure S6 and Figure S7). Based on the full view of the NMR spectrum, the left signal (4.8 ppm) is the signal of the solvent (D_2_O), and the other two main signals belong to LA (see Additional file 1: Figure S6). The NMR results for the study of sample (#14) and can be compared with the NMR spectrum of PGA from the literature (see Additional file 1: Figure S7 and Figure S8). Although the signal intensities are much smaller due to the lower concentration, the typical signal pattern can be identified, such as a split triplet between 1.9 and 2,4 ppm, labeled with A, and a singlet at 4 ppm, labeled with B (see Additional file 1: Figure S7). The impurity in sample (#14) is assigned to PGA.

The sensitivity of the CE-MS system provides a certain assurance of the excludability of the presence of other organic compounds in the LA solution of sample (#14).

### Determination of the PGA concentration

Information on the identity of the critical impurity, PGA, was used to measure the concentration in the LA DSP process and the purified LA samples. Accordingly, the linearity of PGA was validated using the method conditions described in sections (LA DSP benchmarks) and (CE-MS for the identification of the critical impurity). A calibration curve for PGA within the range of 2–1,000 ppm was created. (S)-2-Pyrrolidone-5-carboxylic acid as the stock solution was diluted to eight concentrations, and the peak areas of the eight data points from the calibration curve were subjected to least-squares regression analysis. Every concentration was measured in triplicate, and the average of the PGA peak area was calculated. The slope (0.00671), intercept (−0.00494) and coefficient of determination (0.99965) were calculated. The linearity of the presented method was well within the relevant concentration range.

The limit of determination (LOD) and the limit of quantitation (LOQ) of the analytical technique for PGA were determined through the signal-to-noise ratio (S/N), according to quality guidelines [24]. The LOD was 0.6 ppm, and the LOQ was 2.2 ppm, with an S/N of 9.7. The precision expressed in terms of relative standard deviation (RSD) for three determinations at 2 ppm was 30.62 % each for the S/N, LOD and LOQ.

Based on the data obtained above, the concentration of PGA was calculated; the results are displayed in Table 1 and Fig. 2. The concentration was subsequently determined using the same CE system, but with a different batch of capillaries and BGE; therefore, the concentration can vary up to 20 %.

**Table 1:** Renewable resources and the obtained concentration (C) of LA, the corresponding purity (P) and the bacteria (B) used, *Lactococcuslactis* (1), *Streptococcus sp.* (2), *Lactobacillus coryniformis subsp. Torquens* (3), *Bacillus coagulans* (4), a mixture of bacteria naturally present in sour whey (5), and the ensilage (6)

#	Resource	B	LA		PGA
[−]	[−]	[−]	C [ppm]	P [%]	C [ppm]
1	Cracked Rye	1	713,000	79.0	3.700
2	Lucerne press juice	1	724,800	61.5	2.300
3	Tapioca starch	2	642,300	84.2	1.300
4	Dextrose	3	662,200	79.5	4.000
5	Sweet sorghum press juice	4	589,600	78.5	1.400
6	Pilsner malt	4	285,400	91.8	1.000
7	Sugar beet molasses	4	667,100	83.4	4.700
8	Straw hydrolysate.	4	726,900	75.4	4.200
9	Sugar cane molasses	4	621,100	84.9	2.600
10	Rape extraction meal	4	556,000	43.4	8.000
11	Whey	5	598,600	83.9	2.400
12	Silage juice	6	316,500	93.6	1.200
13	Sugar bread	4	728,200	85.5	2.400
14	Animal bone meal	4	718,400	88.3	0.3
15	Rape extraction meal and molasses	4	627,800	74.6	12.500
16	Purac 88 % w/w	-	669.800	71.2	0.0

### Causes for changing PGA concentrations

**Fig. 4:**
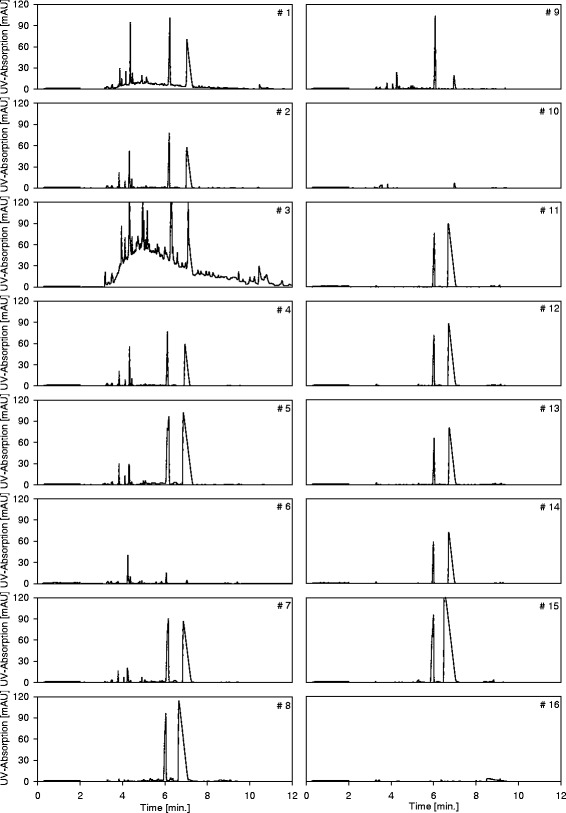
Electropherograms of every unit operation taken from the DSP

PGA was present in all LA solutions, independent of the type of renewable resource or the bacterium applied (Figs. 3 and 4). PGA, also known as 5-oxoproline, is a metabolite in the glutathione cycle, which is present in all living microorganisms [25]. PGA is found in many proteins, and during intracellular protein metabolism, N-terminal glutamic acid and glutamine residues can spontaneously cyclize to become PGA [26]. Hence, the concentration of PGA in the LA solution can only be limited to a certain amount. PGA is converted to glutamate by 5-oxoprolinase, which was successfully separated from the LA solution by EDBM (section (Instrumentations) and Fig. 3). The EDBM is not suitable for a total removal of the PGA from the LA stream, due to its high energy demand and the resulting operational cost. However, the GPA concentration can be reduced by lengthening the residence time in batch operation mode during fermentation (Additional file 1: Figure S9). Our forthcoming work will be on the investigation of the right operation mode in batch and continuous fermentation to yield PGA free LA.

### Determination of the impact of PGA on the LA polymerization process

The EPGs obtained from the industrially produced LA (#16) and the LA produced in the pilot plant facility (#1–15) are shown in Fig. 4. Based on a comparison, four different types of peaks are clearly present. The first fraction of peaks is located between 0 min and 2 min, at 3 min and at 9 min. The lactate peak is located at 6 min 36 s to 7 min. The PGA peak is located at 6 min. However, there are two additional peaks at 4 min 36 s to 4 min 48 s and 5 min to 5 min 36 s.

The first peak fraction originates from the CE method, as they are also present in the EPG of the blank run (#1 in Additional file 1: Figure S10). The appearance of the other two peaks can possibly be explained by the ability of LA to self-condense into di- and trilactide at higher concentrations [27].

This effect is recorded through the EPG of three samples from the DSP. The first sample originates from the DSP after the WBA, immediately before concentration through distillation (#14 in Fig. 3 and #2 in Additional file 1: Figure S10). The second sample was obtained immediately after concentration (#15 in Fig. 3 and #3 in Additional file 1: Figure S10); the same sample was analyzed again after four weeks of storage (#4 in Additional file 1: Figure S10). Comparison of the sample after storage and the industrially produced sample shows the formation of lactide over time (#5 in Additional file 1: Figure S10). The two peaks represent di- and tri-lactide at a specific concentration, and due to their presence, the purity of the industrial LA (#16 in Table 1) was determined to be 71.2 %. As those peaks do not represent organic impurities that change color or disrupt the polymerization process, PGA is the only relevant organic impurity found in the DSP that could disrupt the polymerization reaction of LA into PLA.

As a carboxylic acid, PGA can in theory react with LA during pre-polycondensation, which would results in a termination of chain elongation during polycondensation [28, 29]. This again can reduce the yield of lactide during de-polymerization [30, 31]. A rapid feasibility test for the polymerization ability of LA was performed by Uhde-Inventa-Fischer Company (UIF, Berlin, Germany). The test allows for the immediate prediction of the commercial usability of a LA solution for polymerization at an industrial scale; however, due to company policy, no details can be revealed about the test. The benchmarks for this test are the lactide yield and the degree of racemization. The degree of racemization for the PLA-process is considered good below 3.0 % and bad above 5.0 %.

Two solutions of the industrial LA containing 5,000 ppm and 0.5 ppm of PGA were investigated in the quick test. These two PGA concentrations represent the lowest and the average amount of detected PGA concentration in the LA solutions (Table 1). For the 0.5 ppm solution, the lactide yield and degree of racemization were 96.2 % and 2.8 %, respectively, and those for the 5,000 ppm solution were 96.3 % and 3.7 %, respectively. The presence of PGA did not affect the amount of yield but did affect the degree of racemization. Based on these results, only limited commercial utilization of a LA solution containing PGA at 5,000 ppm can be realized.

PGA proved to be a critical impurity according to all evaluations. Due to its natural occurrence during cellular metabolism, it is omnipresent in every fermentation process. In our study, PGA successfully resisted separation from the LA solution by the DSP and disrupted, even in comparatively small concentrations, the polymerization reaction. Further studies need to be conducted with regard to the efficient separation of PGA from the LA solution. Initial results for a partial separation by EDBM are already presented in this study (Fig. 3).

## Conclusions

The present study proved the CE system to be an important tool for DSP monitoring. The high product concentration encountered in biological production processes did not hinder the CE from separating and detecting organic impurities, even at minor concentrations. The coupling of the CE with an MS system allowed for the straightforward identification of the remaining critical impurity, PGA. Although 11 separation units were applied during the DSP, the PGA concentration remained at 12,900 ppm, which was comparatively high. All organic impurities found were tracked by the CE, allowing for further separation optimization.

## Methods

### Instrumentations

Electropherograms were recorded with the Agilent 7100 Capillary Electrophoresis System (Agilent Technologies Deutschland GmbH, Böblingen, Germany) using an UV–VIS detector. The system was controlled with a computer equipped with Agilent Technologies OpenLAB CDS ChemStation Edition software for data collection and analysis. All bare fused-silica capillaries (50 μm i.d.) used in these experiments were purchased form Agilent Technologies Deutschland GmbH (Böblingen, Germany). The capillaries were 64 cm in length and equipped with a bubble cell. All samples were measured in triplicate, and the mean concentration is reported. All samples and buffers were previously filtered through a 0.45 μm pore size polyvinylidene fluoride (PVDF) membrane (Bio-Rad Laboratories GmbH, Munich, Germany) and degassed by centrifugation at 21913 g for 3 min.

The identification of unknown impurities within the DSP that could not be identified through spiking the CE-UV/VIS system was realized with the advanced setup of CE-MS. All experiments were performed with a lab-built automated CE system and a time-of-flight (TOF) mass spectrometer (BrukerDaltonik, Bremen, Germany) equipped with a sheath-liquid electrospray ion source (Agilent, Waldbronn, Germany). The system was controlled with a computer using micrOTOF control software, version 1.3, from BrukerDaltonik. Compass Data Analysis software, version 4.0, from BrukerDaltonik was used for data analysis. The software for the CE-system was designed and developed with LabView 2009.

### Electrophoretic procedures and conditions

The BGE for CE-UV/VIS was prepared daily with 25 mM borate and 50 mM sodium dodecyl sulfate (SDS). New capillaries were preconditioned with 0.1 N NaOH for 3 min, rinsed with ultrapure water for 1 min and conditioned with the buffer for 5 min. Afterwards, 5 blank runs were processed to establish constant migration times. Before each run, the capillary was flushed with BGE for 3 min at 0.1 MPa. The entire sampling sequence consisted of 1 blank run, 1 electrolyte run, between 1 and up to 10 runs of samples, 1 electrolyte run, and 1 blank run.

Sample injection was carried out in hydrodynamic mode with a time of 5 s, a pressure of 50 mbar, a current of −30 kV and a temperature of 35 °C. The detection mode was direct detection at a wavelength of 200 nm. The mirroring of the negative peaks along the x-axis generated a more familiar electropherogram for integration and processing.

For the CE-MS analysis, a BGE consisting of 25 mM ammonium acetate adjusted to pH 8.6 with ammonia was used. The chemicals ammonium acetate and ammonia were purchased from Merck (Darmstadt, Germany) and were used as received. The sheath-liquid consisted of water, isopropanol and ammonia in a ratio of 49.9:49.9:0.2. LA and pyroglutamic acid were purchased from Sigma-Aldrich Chemie GmbH (Taufkirchen, Germany). Fused-silica capillaries (25 cm length, 25 μm i.d.) were purchased from Polymicro Technologies (Phoenix, AZ, USA).

Industrial grade 88.0 % (w/w) L(+)-LA, chemical name 2-hydroxypropionic acid, was purchased from Purac (Bingen am Rhein, Germany) [32]. (S)-2-Pyrrolidone-5-carboxylic acid was purchased from Merck (Darmstadt, Germany) for the CE-UV/VIS analysis.

### USP of renewable resources into LA

Table 1 shows the 15 different renewable resources that were converted into LA. Although the volume of the auxiliary chemicals and enzymes changed according to the applied resource material, the USP procedure remained the same. The description is given at the example of our main process discussed in this study below. This USP can be separated into three parts.

1. Preparation of the raw feed solution was carried out by mixing 50 L of deionized water with the 7.5 kg of rapeseed in a 75 L vessel BIOSTAT UD (Sartorius AG, Goettingen, Germany) equipped with a cooling and heating jacket. A pitched-blade impeller with 3 × 6 blades was used for the agitation process. The stirring velocity was set to 200 rpm. The temperature was set to 119 °C for 30 min and then set to 25 °C for 24 h. The pH level was set to pH 6.0 by the addition 5.55 mL 20 % NaOH. After 24 h the pH of the raw feed solution was adjusted again to pH 6.0 by the addition of another 71 mL 20 % NaOH, afterwards the raw feed solution was inoculated in the same vessel according to step 2.
2. The hydrolysate was prepared by adding 11.25 mL *Flavourzyme*, 11.25 mL *Neutrase*, and 5 mL *Viskozym* (Novozymes, Bagsværd, Denmark) at 50 °C to the raw feed solution and stirring the solution for 6 h. The agitation conditions were the same as in step 1.
  The hydrolysate was thermally sterilized at 121 °C and 2 bar for 20 min. Coarse particles were removed by a filter bag (200 μm) and a ultra filtration device (0.1 μm) 4 × ABB (ABB Limited Process Automation pumping, Mannheim, Germany) The filtered and sterilized hydrolysate was then fed into the fermentation process.
3. Fermentation in continuous mode was carried out using a 5 L fermenter. Cell retention was established by pumping the fermentation broth over a polyvinylidene fluoride hollow fiber membrane module with a pore size of 0.2 μm and a membrane area of 0.09 m^2^ UMP-1047R Pall (Dreieich, Germany). Preparation of the microorganisms was established by the addition of colonies taken from three tubes with tilted agar and the addition of 180 mL de Man, Rogosa and Sharpe growth medium (MRS) and 2 g EVERZIT Dolomit 0,5–2,5 mm EVERS (Hopsten, Germany) in a flask shakers. The obtained mixture was shaken at 100 rpm at 52 °C for 15 h. The ratio of microorganisms to broth was 0.06 with 180 mL in the 5 L vessel.

The bacterial strains shown in Table 1 were added, and the temperature was set to 52 °C. The temperature was maintained by a cooling jacket for 69 h. An additional amount of deionized water was added at the beginning of the fermentation process, and during the process, 20 % NaOH was continuously added to maintain a pH of 6.0. The exponential growth was reached on average after 8 h. For the continuous fermentation flow rates of 0,1 L/h were applied, with an addition of 2,5 mL/min hydrolysate and 2,5 mL/min molasses. The concentration of the molasses feed increased from the beginning of the exponential growth phase 178 g/L (8 h) to 222.5 g/L at 31 h and ending at 244.7 g/L (80 h).

The batch fermentations were started by the addition of 1.5 L hydrolysate and 1.5 L molasses at a concentration of 89 g/L without the addition of any further salts.

The fermentation broth was collected and sterilized at 121 °C and 2 bar for 20 min.

### DSP of fermentative-produced LA

The fermentation broth was collected and purified according to the DSP illustrated in Fig. 1. The DSP includes eleven separation units, which are arranged as follows.

Two filtration units equipped with micro-(MF) (#1) and nanofiltration (NF) (#2) membranes were installed at the beginning of the DSP. The thin film NF membrane has a molecular weight cut off of 150–300 g mol^−1^ for uncharged molecules (DL2540F1073, GE).

An acid-chelating resin with a macroporous polystyrene cross-linked polymer and divinylbenzene and aminophosphonic functional groups was utilized after the filtration process [33](S950, Purolite). The acid-chelating resin was applied before (Softening 1) (#4) and after (Softening 2) (#7) the monopolar electrodialysis (ED) process.

Two types of membranes were installed: ED and bipolar electrodialysis (EDBM). Both electrodialysis units were equipped with the same type of anion (Fumasep FAB) and cation (Fumasep FKB) exchange membranes (FuMA-Tech GmbH, Bietigheim-Bissingen, Germany) but in a different configuration [34].

After the EDBM (#8), two decolorization (DC) steps (#11 and #12) were carried out using a strong acid-adsorbent resin with divinylbenzene and sulfonic acid functional groups (MN502, Purolite). The designed application was the sorption and separation of hydrophobic organic species onto its cationic matrix [33].

After the DC, a strong acid cation (SAC)-exchange resin (#13) with sulfonic functional groups was applied. This allowed for applications in oxidizing media in the presence of inorganic salts and organic nitrogen-containing compounds such as amino acids, peptides and proteins [35].

After the SAC, a weak basic anion (WBA)-exchange resin (#14) with tertiary amine functional groups was applied; the high hydrophobic character allowed for the processing of organic acids [35]. Both ion exchange resins have a highly porous copolymer styrene-divinylbenzene matrix (Resindion s.l.r., Binasco, Italy).

The final concentration step was evaporation (#15), which removed excess water, yielding concentrated LA.

## Additional file

Additional file 1: Supporting Information. (DOCX 268 kb)

## Abbreviations

B: bacteria
BGE: background electrolyte
C: concentration
CE: capillary electrophoresis
DC: decolorization
DSP: downstream process
ED: electrodialysis, monopolar
EDBM: electrodialysis, bipolar
LA: lactic acid
LOD: Limit of determination
LOQ: limit of quantitation
MF: microfiltration
MS: mass spectrometry
NF: nanofiltration
NMR: nuclear magnetic resonance spectroscopy
P: purity
PGA: pyroglutamic acid
PLA: poly lactic acid
PVDF: polyvinylidene fluoride
S/N: signal-to-noise ratio
SAC: strong acid cation
SDS: sodium dodecyl sulfate
TOF: time-of-flight
USP: upstream process
UV/VIS: ultraviolet and visible spectroscopy
WBA: weak basic anion
