# Supplementary material for: CE-UV/VIS and CE-MS for monitoring organic impurities during the downstream processing of fermentative-produced lactic acid from second-generation renewable feedstocks
Source: J Biol Eng. 2016 May 19;10:7. doi: 10.1186/s13036-016-0027-2 (PMC4872333; doi:10.1186/s13036-016-0027-2)
Supplement: Additional file 1: — Supporting Information. (DOCX 268 kb) [file 13036_2016_27_MOESM1_ESM.docx]

**Additional file 1: Supporting Information**

**CE-UV/VIS and CE-MS for the monitoring of organic impurities during downstream processing of fermentative produced lactic acid from second generation renewable feedstocks**

Hendrik Laube^1*^, Frank-Michael Matysik^2^, Andreas Schmidberger^2^

Kerstin Mehlmann^1^, Andreas Toursel^1^, Jana Boden^3^

^1^Department of Bioengineering, Leibniz-Institute for Agricultural Engineering (ATB), Max-Eyth-Allee 100, Potsdam 14469, Germany

^2^Institute of Analytical Chemistry,Chemo- and Biosensors,University of Regensburg,Regensburg, Germany

^3^ICA Boden-Haumann-Mainka, Engineering society for chemical analysis, Langen, Hessen, Germany

**Corresponding author** Tel.: +49 (0) 331 5699 121; fax: +49 (0) 331 5699 849

E-mail address: [hlaube@atb-potsdam.de](mailto:hlaube@atb-potsdam.de) (H. Laube)

# Tables and Figures Capitations

**Supplementary Table 1.** Possible molecular formulas for the unknown compound calculated with a software tool (SmartFormula). Given parameters: molecular formulas, score, measured m/z ratio, error in mass (mDa and ppm), and the fit of measured and calculated isotopic pattern (mSigma).

**Supplementary Figure 1.** Extracted ion electropherogram of the diluted sample No. 14 (1:5) based on CE-MS measurements. BGE: 25 mM ammonium acetate, pH 8.6. Capillary (25 cm length, 25 µm i.d.). Black: unknown compound. Red: lactic acid. The separation voltage was 30 kV.

**Supplementary Figure 2.** Extracted mass spectrum of the separated impurity in the lactic acid sample No. 14.Left: Mass peak of the impurity.

**Supplementary Figure 3.** Isotopic pattern of unknown compound in the sample #14 (top) and the calculated isotopic pattern for the formula C_5_H_6_NO_3_.

**Supplementary Figure 4.** Electropherograms of the impurity m/z 128.042 in the diluted sample No. 14 and of the 1 mM PG-standard solution (in lactic acid matrix). BGE: 25 mM ammonium acetate pH 8.6. Capillary (25 cm length, 25 µm i.d.). Black: PGA-standard 1 mM. Red: unknown compound. Separation voltage 25 kV.

**Supplementary Figure 5.** Extracted mass spectrum of the PGA standard solution. Left: Mass peak of the PGA standard.

**Supplementary Figure 6.** Full view of the NMR-spectra of the sample #14.

**Supplementary Figure 7.** Part of the spectrum where the signals of the unknown compound can be seen. Regions of interest labeled with A and B. Typical signal pattern for the LA can be identified, such as a splitted triplet between 1.9 and 2.4 ppm, labeled with A and a singulet at 4 ppm, labeled with B.

**Supplementary Figure 8.** Experimental NMR spectrum of pyroglutamic acid. (Source: scifinder.cas.org). The labels A and B correspond to the same signals as in Fig. 7.

**Supplementary Figure 9.** a) Optical Activity (value linked to the amount of biomass in the fermenter) of PGA during a 3 L and 100 L continuous fermentation

b) Optical Activity (value linked to the amount of biomass in the fermenter) of glucose during a 3 L and 100 L continuous fermentation

For further information please consult the Metabolomics research project found at:<http://www.atb-potsdam.de/en/research-programs/project.html?xq=273>

**Supplementary Figure 10.** EPG taken from the blank run (#1), sample from the DSP after the WBA (#14) (#2), sample from the DSP after the distillation (#15) (#3), same sample as (#15) after a storage of 4 weeks (#4) and the Purac industrial LA (#5).

**Supplementary Figure 11.** Identified impurities peaks from the spiking experiments.

| Supplementary Table 1 | | | | | | | |
| --- | --- | --- | --- | --- | --- | --- | --- |
| # | Formula | Score | m/z | Error | Error | mSigma | rdb |
| [-] | [-] | [-] | [-] | [mDa] | [ppm] | [-] | [-] |
| 1 | C_5_H_6_NO_3_ | 1.98 | 128.03532 | -6.9 | -53.8 | 8.1 | 3.5 |
| 2 | C_4_H_6_N_3_O_2_ | 100.00 | 128.04655 | 4.3 | 33.9 | 9.6 | 3.5 |
| 3 | CH_2_N_7_O | 0.00 | 128.03263 | -9.6 | -74.6 | 19.5 | 4.5 |
| 4 | C_9_H_6_N | 0.03 | 128.05057 | 8.4 | 65.4 | 58.4 | 7.5 |
| 5 | C_7_H_2_N_3_ | 0.00 | 128.02542 | 0.0 | 0.0 | 781.9 | 8.5 |

| 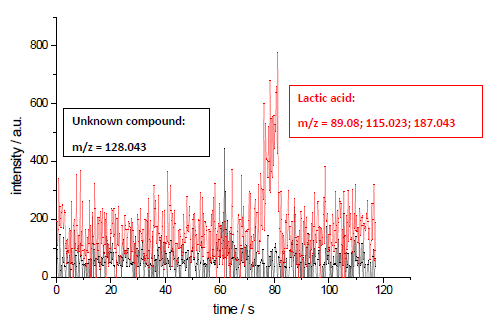 |
| --- |
| Supplementary Figure 1 |

| 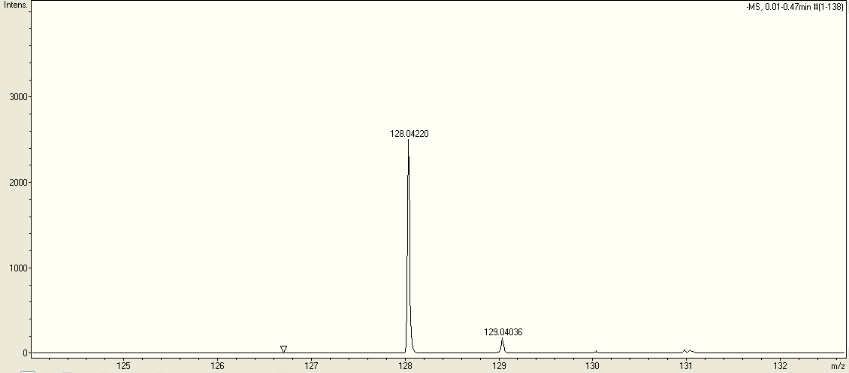 |
| --- |
| Supplementary Figure 2 |

| 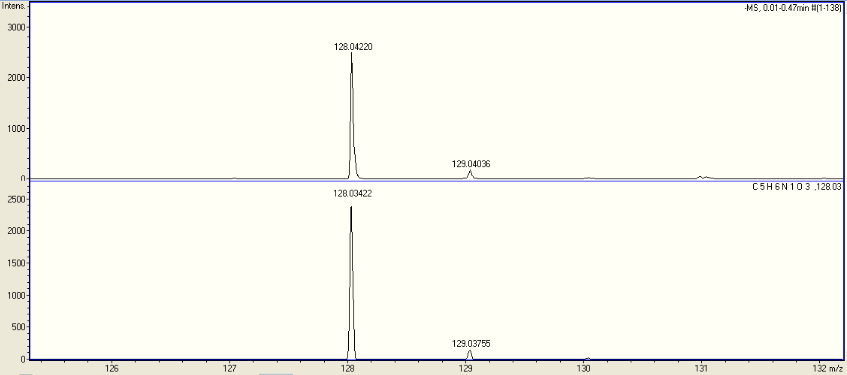 |
| --- |
| Supplementary Figure 3 |

| 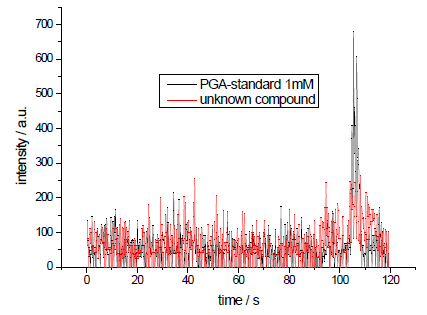 |
| --- |
| Supplementary Figure 4 |

| 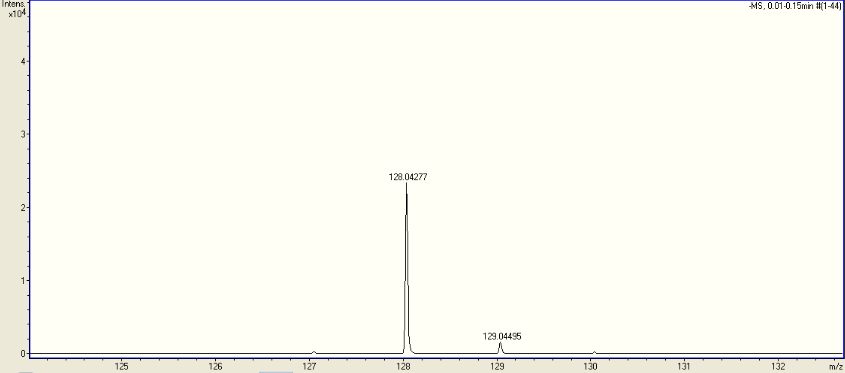 |
| --- |
| Supplementary Figure 5 |

| 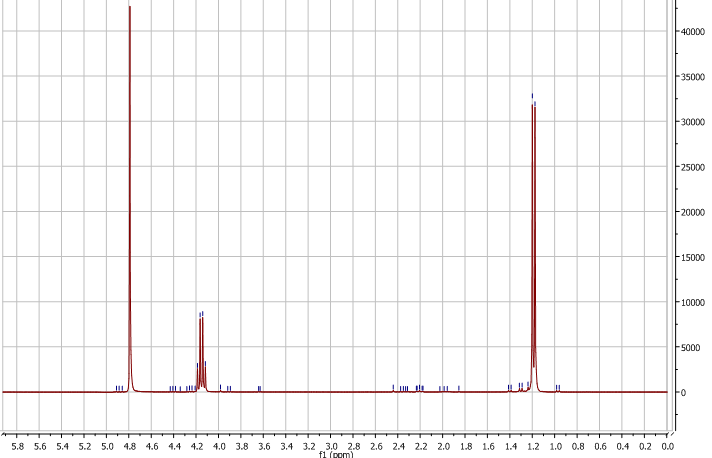 |
| --- |
| Supplementary Figure 6 |

| 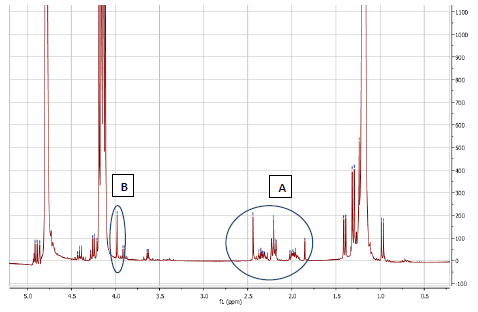 |
| --- |
| Supplementary Figure 7 |

| 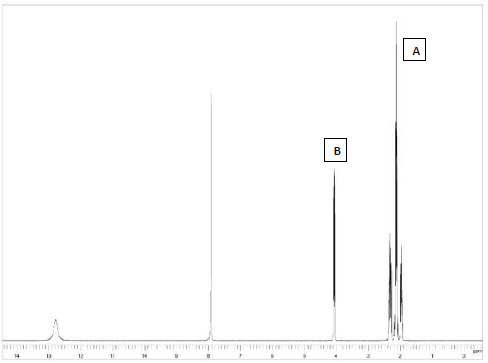 |
| --- |
| Supplementary Figure 8 |

|  |
| --- |
| a) |
|  |
| b) |
| Supplementary Figure 9 |

|  |
| --- |
| Supplementary Figure 10 |

|  |
| --- |
| Supplementary Figure 11 |
